# Lessons learned from implementation of the Workload Indicator of Staffing Need (WISN) methodology: an international Delphi study of expert users

**DOI:** 10.1186/s12960-021-00675-z

**Published:** 2022-01-28

**Authors:** Grace Nyendwoha Namaganda, Audrey Whitright, Everd Bikaitwoha Maniple

**Affiliations:** 1 Chemonics International, Human Resources for Health in 2030 Program, 191/14 Petroda House, Presidential Way, Lilongwe, Malawi; 2 School of Nursing and Health Sciences, Capella University, Minneapolis, MN 55402-4319 USA; 3 Kabale University, Plot 364 Block 3 Kikungiri Hill, Kabale Municipality, Uganda

**Keywords:** WISN, Health workforce, Staffing levels, Traditional Delphi, Planning, Health systems research

## Abstract

**Background:**

Staffing of health services ought to consider the workload experienced to maximize efficiency. However, this is rarely the case, due to lack of an appropriate approach. The World Health Organization (WHO) developed and has promoted the Workload Indicators of Staffing Need (WISN) methodology globally. Due to its relative simplicity compared to previous methods, the WISN has been used extensively, particularly after its computerization in 2010. Many lessons have been learnt from the introduction and promotion of the methodology across the globe but have, hitherto, not been synthesized for technical and policy consideration. This study gathered, synthesized, and now shares the key adaptations, innovations, and lessons learned. These could facilitate lesson-learning and motivate the WHO’s WISN Thematic Working Group to review and further ease its application.

**Methods:**

The study aimed to answer four questions: (1) how easy is it for the users to implement each step of the WISN methodology? (2) What innovations have been used to overcome implementation challenges? (3) What lessons have been learned that could inform future WISN implementation? and (4) what recommendations can be made to improve the WISN methodology? We used a three-round traditional Delphi method to conduct a case study of user-experiences during the adoption of the WISN methodology. We sent three email iterations to 23 purposively selected WISN expert users across 21 countries in five continents. Thematic analysis of each round was done simultaneously with data collection.

**Results:**

Participants rated seven of the eight technical steps of the WISN as either “very easy” or “easy” to implement. The step considered most difficult was obtaining the Category Allowance Factors (CAF). Key lessons learned were that: the benefits gained from applying the WISN outweigh the challenges faced in understanding the technical steps; benchmarking during WISN implementation saves time; data quality is critical for successful implementation; and starting with small-scale projects sets the ground better for more effective scale-up than attempting massive national application of the methodology the first time round.

**Conclusions:**

The study provides a good reference for easing WISN implementation for new users and for WHO to continue promoting and improving upon it.

**Supplementary Information:**

The online version contains supplementary material available at 10.1186/s12960-021-00675-z.

## Background

The 2006 World Health Report “Working Together for Health” recognized human resources for health (HRH) as the most important resource in the delivery of health services [1]. This was followed by a wide range of innovations aimed at ensuring efficient utilization of the available HRH given the widespread shortages. Globally, health systems face escalating health care costs requiring implementation of cost-containment measures to ensure the maximum possible population health within the available resources, including HRH [2–6]. However, determining the right number of health workers, with the right skills, in the right place, at the right time, to provide the right services, is a major challenge affecting health systems especially in less developed countries [7, 8]. This is partly due to lack of easy-to-use planning methods and tools that are responsive to the unique challenges faced by managers within health systems of those countries [9]. Extant literature is replete with approaches for determining HRH requirements, such as health worker-to-population ratio, utilization and demand approach, service target approach, and health service needs approach, among others [6, 7, 10–12]. However, those methods have significant drawbacks that make them unsuitable for use by most developing countries.

In the search for an ideal method of determining HRH requirements, Tomblin Murphy et al. [6] developed criteria to guide countries in defining the most appropriate method. The criteria included ensuring that the method: (1) is consistent with the objectives of the country’s health care system, (2) derives HRH requirements from service requirements, (3) considers service productivity by the different types of HRH, (4) measures HRH availability in terms of their actual time devoted to service delivery, (5) considers the factors that affect hours worked, (6) considers the implications of HRH plans and their alignment to the health system’s financial plans, and (7) considers HRH requirements in the broader health care service production context. It is not surprising, therefore, that finding the ideal method is difficult. However, the WHO’s Workload Indicators of Staffing Need (WISN) methodology meets most of the criteria of the ideal method.

The WISN helps to determine HRH needs based on the workload experienced by an individual health facility. It is consistent with the objectives of the country’s health care system and considers the number and type of health workers currently available for work, the time that they are available for work, and the amount of work each one can accomplish during this available time. It helps to estimate the cost implications of the additional HRH and can inform health sector financial plans. It is comparatively simpler to use than earlier methods of determining HRH requirements that are complex and require enormous data [13–16]. The WISN’s underlying assumptions are technically sound and uses routinely collected data, making it simple and easy to understand [10, 12, 13]. However, it does not directly estimate HRH requirements as a function of population health measures.

Due to its relative simplicity, the WISN methodology has been used extensively to inform staffing decisions, especially since its computerization in 2010 [5, 13, 17, 18]. In the process, a wealth of experience has been gained which, unfortunately, is scattered across the countries and has neither been well documented nor synthesized into lessons to promote peer learning. This study was conducted to gather, synthesize, and share such implementation experiences with a view to promote learning across countries and health systems. It identified and documented key challenges and solutions and synthesized the lessons learned. These now form a body of knowledge that could facilitate further innovation and learning among WISN users and motivate the WHO’s WISN Thematic Working Group to review the methodology and further ease its application. In its current form, the WISN has eight technical steps: determining priority cadres and health facility types; estimating available working time; defining workload components; setting activity standards; establishing standard workloads; calculating allowance factors; determining staff requirements based on WISN; and analyzing and interpreting WISN results. An even easier WISN will further spur its widespread use to aid evidence-based workforce planning.

## Methods

The study aimed to answer four questions:

1. How easy is it for the users to implement each of the eight technical steps of the WISN?
2. What strategies have been used to overcome implementation challenges?
3. What lessons have been learned by the users that could inform improvements on the design of the WISN methodology and help future studies?
4. What recommendations can be made to improve the WISN methodology and ease its technical implementation?

Data were collected using the Traditional Delphi method [19–22] through anonymous interactions and controlled feedback via email with WISN expert users drawn from 21 countries. Those included in the panel discussions were selected purposively from a wider pool of WISN users and had to meet all of the following eligibility criteria:

- Conducted a WISN assessment using the revised 2010 WISN User’s Manual [13],
- Directly implemented at least 75% (6/8) of the WISN technical steps,
- Conducted more than one small scale WISN study or at least one large scale WISN study,
- Willing to commit at least 3 h to the study,
- Committed to provide honest responses during the Delphi discussions,
- Willing to participate in the Delphi discussions via email, and
- Willing to participate in the Delphi discussions in English.

A total of 52 expert users were identified from published WISN literature and recommendations by peers and 23 of them met all the eligibility criteria. Wilkes [20] asserts that for a homogenous Delphi sample, 10–15 panelists are adequate. However, this study included all the 23 to cater for possible dropouts along the way.

### Data collection

Data were collected in three rounds of Delphi discussions via email using three field-tested questionnaires [21, 23] approved by the first author’s institutional review board. Anonymity was attained by use of a third party to send out the questionnaires, receive responses, assign a unique code to each expert, securely store the data, and to strip the data of all personal identifiers before submitting them to the first author.

In the first-round, the experts were asked to individually assess how easy it was for them to implement each of the WISN technical steps, on a five-point Likert scale of: “very easy”, “easy”, “neither easy nor difficult”, “difficult”, and “very difficult”. They were also asked to highlight the key enabling factors and difficulties experienced in implementing the steps, the strategies/innovations they had used to address the difficulties encountered, the lessons learned, and to make recommendations to improve the WISN. All responses were submitted to the third party as Microsoft Word scripts and later submitted to the first author for analysis after de-identification. The findings from the first round were used to develop the questionnaires for subsequent rounds, as in previous Delphi studies [23].

In the second round, a five-point Likert scale of: “strongly agree”, “agree”, “neither agree nor disagree”, “disagree”, and “strongly disagree”, was used to obtain consensus on key enabling factors in using the WISN methodology. A five-point Likert scale of: “very effective”, “effective”, “moderately effective”, “slightly effective”, and “not effective”, was used to obtain consensus on the perceived effectiveness of the strategies/innovations they had used to address challenges. The third and final round questionnaire sought to obtain consensus on key lessons learned, recommendations for easing implementation of the WISN technical steps, and recommendations for improving the methodology. Only those strategies/innovations considered to be “effective” by the experts during the second round were used to formulate the final lessons and recommendations.

### Data analysis

Thematic inductive analysis [24] was used to analyze the qualitative data. Codes were allocated to data sections with specific meanings. Key patterns of meanings were identified and grouped into themes which were, then, used to formulate the subsequent questionnaires. Quotes highlighting unique and vivid experiences were identified and are presented.

## Results

The 23 eligible WISN expert users were from 21 countries across five continents. Response rates for each round of Delphi discussion were high, at 100% (23/23) for the first and second rounds and 91% (21/23) for the third round. Most of the respondents worked in the field of HRH, public health, and/or academia. Most (91% or 21/23) had directly implemented all the WISN technical steps and 65% (15/23) had conducted large scale WISN studies. 50% of the respondents had conducted two or more WISN studies, while the other 50% had conducted only one WISN study. In addition, 61% of the respondents had more than 2 years of WISN experience, while 39% had 2 years of experience or less.

### Ease of implementation of each WISN technical step

Summary results on this question are presented in Table 1 while the detailed results are in Additional file 1. Seven out of the eight of the WISN technical steps were rated as either “very easy” or “easy”. Calculating and explaining the rationale of the category allowance factor (CAF) was highlighted as the only challenging step. The difficulties experienced in implementing each technical step are summarized in the last column.

**Table 1:** Practicability of implementing each WISN technical step

No	Technical step	Practicability consensus	Key difficulties encountered
1	Determining priority cadres and health facility types	Very easy	Negotiating a feasible study scope
2	Estimating available working time (AWT)	Easy	Inadequate data on staff absences Actual AWT lower than that from WISN due to late coming and unofficial absenteeism
3	Defining workload components	Easy	Task-shifting and task sharing complicate the process Difficulty gaining consensus on which main workload components to include in the study
4	Setting activity standards	Easy	The process needs a lot of time Relying only on expert group discussions to set activity standards is subjective Due to task-shifting experts tend to set activity standards for what the staff are currently doing and not what they should be doing
5	Establishing standard workloads	Easy	None
6	Calculating allowance factors (CAF)	Easy with mid-point	Explaining the CAF concept is difficult and CAF formula is intimidating
7	Determining staff requirements	Easy	Poor data quality—missing, incomplete, or data not easily accessible Different data systems and reporting formats in every system Data collection process is time consuming Data entry into the software is labor intensive, since data are entered one facility at a time
8	Analyzing and interpreting WISN results	Easy	Ensuring results are accepted and implemented is difficult Lack of policy supporting use of WISN is a key barrier

The experts highlighted several factors that they felt enabled implementing the WISN: (1) pre-implementation training of the different groups involved; (2) automation of the methodology; (3) provision of formulas which make the calculations easy and reduce errors; (4) the WISN User’s Manual which clearly explains how each WISN technical step is to be implemented; (5) senior-level leadership support ensuring collective problem-solving during implementation; and (6) good health information systems ensuring easy access to reliable workload and staffing data.

### Strategies/innovations used to overcome implementation challenges

The key challenges reportedly encountered in implementing the WISN and the strategies/innovations used to mitigate them are summarized in Table 2. Key innovations highlighted by the WISN experts included automating data entry to minimize laborious data entry processes, benchmarking on other countries to reduce the time spent on setting activity standards, and setting activity standards for bedside nursing to accurately determine the inpatient workload and requirements for the nursing cadres.

**Table 2:** Challenges experienced and strategies/innovations used

No	Challenge	Strategy/innovation to mitigate challenge
1	Poor data quality and several data sources/systems	Used available data to extrapolate annual workload using different approaches or carried out primary data collection Benchmarked on other countries to address inaccurate or missing data on staff absences coupled with expert group discussions Triangulated the data from different sources
2	Laborious data entry	Locally developed software to export data into the WISN Software Enabled server-based data entry for simultaneous data entry by a large team of data entrants Prepared the data outside the WISN software before data entry and imported them later
3	Widespread task-shifting	Defined activities based on what each cadre should be doing and not what they are doing, where there was no task-shifting policy Apportioned the shared workload to the different cadres based on how much (percent) each cadre does and used this to determine the staffing requirement for each of the cadres
4	Actual working time shorter than AWT	Highlighted this as a management problem that needs to be addressed through strengthened supervision and management
5	Ensuring use of WISN results	Involved key stakeholders from the start of WISN implementation Held consultative workshops to collectively formulate recommendations based on study results Provided several scenarios for using the results Phased approach to implementation of recommendations Advocating for a policy change from fixed to workload-based staffing norms
6	Setting activity standards: described as time consuming and complicated example, e.g., bedside nursing activities did not have a clear workload statistic	Ensured that the expert group had the required level of expertise, experience, and credibility Allowed adequate time for discussion and debate within the expert group Benchmarked from other countries to save time Ensured tasks per cadre were in line with what the cadre *should do* and not what they *are doing* currently Set bedside nursing activity standards that varied by patient acuity with patient days as the workload statistic

The bedside nursing strategy was further described thus by one of the WISN experts:

*Much of the client-level work of nurses is not accounted for by any service statistics … we did a time-motion study on a sample of facility types to identify how much time the different nursing activities took to come up with an activity standard. The hospital-level WISN results for nurses were more reliable after developing this activity standard ****[Expert 1 with 5–10 years using WISN].***

### Lessons learned to inform future WISN studies and improvements of the WISN methodology

The WISN experts shared several lessons learned based on their implementation experience and these are presented in Table 3.

**Table 3:** Key lessons learned

#	Lesson learned	Key highlights of the lesson learned
1	The WISN process is more important than the technical steps, because throughout the process, a lot is learned on how to better plan and manage the workforce and it promotes consensus at each stage and hence ownership	Careful design and implementation of the WISN process increases chances of using the results Allocate ample time for the key WISN processes and ensure consensus at each stage Involve key stakeholders early in the process Obtain senior level management support for the WISN A bottom up WISN implementation process is motivating to staff
2	The WISN is not a panacea for all HRH issues	WISN is not a solution to all HRH challenges There is need to address other HRH issues, such as: - Performance including absenteeism, - Competencies, - Attraction, motivation, and retention and, - Supportive supervision, among others
3	Implementation of the technical steps of the WISN methodology requires specific competencies	Reading the WISN User’s Manual alone is not enough Key competencies required include: - Good understanding of the health sector - Good mathematical skills - Good computer skills - Flexibility - Good understanding of the local context if possible - Previous experience practically implementing the WISN or collaborating with an experienced WISN user
4	Build on previous WISN experiences	Review what others have done and build on that—it saves time Benchmark in determining workload components, setting activity standards, and determining AWT Collaborate with an experienced WISN user
5	Good quality data in terms of completeness, accuracy, and availability are critical for successful implementation of WISN	Without good quality data, the WISN is impossible to implement accurately
6	WISN practical implementation is more complicated than described in the Manual	Implementation in secondary and tertiary hospitals is more complicated than described in the WISN User’s Manual hence the need for segmentation of the guidance to address the needs of the different levels of the health system The Manual needs to provide more guidance on how to address task-shifting, prepare the data before entry into the software, make data entry less laborious, and how to set activity standards for bedside nursing The Manual needs to provide more examples and tools to manage the different WISN technical steps
7	Start small	Start with small WISN studies before implementing larger studies: for example, starting at individual hospital level, then district level, then regional level, before national/health system-wide level to enhances skill, build confidence in the methodology, and ensure that the approach is feasible The small wins during implementation of small studies are motivating
8	Setting activity standards is subjective	Using only expert group discussions to set activity standards is subjective Complement the expert group discussions with other methods, such as direct observation, benchmarking, time motion studies and role-playing Consider validating standards set by one expert group with another
9	WISN study recommendations may have significant financial implications	Resources may be need for: - Recruiting additional health workers - Training additional health workers - Addressing other retention challenges, such as staff accommodation, training, resettlement packages, and transport if they must transfer staff across facilities, among others

The experts highlighted the importance of the WISN process, particularly stakeholder involvement and consensus-building at each technical step that, in their view, ensures ownership and use of results:

*The WISN process is more important than the actual steps of implementing the technical part, and the use of WISN in decision making is only possible if the process has been carefully designed and implemented, ensuring that all stakeholders are on board ****[Expert 2 with 5–10 years using WISN].***

### Experts’ recommendations to improve the methodology and ease WISN technical implementation

Based on their experience in implementing the WISN technical steps, the experts made several recommendations to improve the methodology and make its technical implementation easier as outlined in Table 4.

**Table 4:** Summary recommendations for enhancing the WISN methodology

No	Recommendation	Specific recommendations
1	Review the WISN software to	Permit direct data importation from other information systems Create an option of server-based data entry Include data analysis functions Capture qualitative data to enable users to explain the results Make the software open source so that it can be further developed and easily customized by users Enable the software to project future workload as an “add-in” to the existing software (version 2.2.170.1) to promote widespread use
2	Review the WHO’s WISN User’s Manual to include	Guidance on how to conduct WISN assessments in secondary and tertiary hospitals Description of how to set activity standards for bedside nursing activities More practical examples for each of the WISN technical steps Practical tools and templates for example, checklists for collecting data, and interview guides for the expert group discussions Examples of activity standards, AWT, and workload components used in other WISN studies Guidance on how to determine staffing requirements for support, administrative, and community health workers A section on how to advocate for the use of WISN results
3	Review the WISN training	Include mini/pilot studies in the training for the technical team to prepare them for the complexities of WISN implementation
4	Advocate for the WISN methodology	Conduct and widely disseminate impact assessment studies linking WISN to improved quality of care and health outcomes Share experiences how the WISN methodology was institutionalized in some countries and its impact
5	Advocate for improvements of data systems	Strengthen existing systems to ensure accurate data Establish a system for tracking staff absences

The experts also advised those planning to conduct a WISN assessment to secure leadership support before they start the process of developing standards, and to start off with small-scale commitments that deal with a few staff categories and facilities. This allows them to develop skills, gain confidence, and build on initial success before scaling up.

## Discussion

The main purpose of this study was to learn from WISN implementation experiences so as to find ways of easing its implementation across countries. The results show that many of the technical steps of the methodology are considered easy to implement, though the degree of ease may vary with the experience of the users and the local context. Resolving the remaining difficulties could lead to its widespread adoption and use across health systems, leading to global evidence-based health workforce planning.

### Lessons from the WISN implementation experiences

Some of the implementation challenges such as difficulties in setting activity standards, laborious data entry process, and difficulty in ensuring the use of WISN results can be mitigated by drawing on the lessons mentioned above. For example, WISN users consider setting activity standards using only expert group discussions to be subjective and recommend the use of mixed methods in setting activity standards. In Botswana, Uganda, Malawi, Namibia, Ghana, and Brazil, such expert group discussions were supplemented with direct observations, motion studies, intra- and intercountry benchmarking, and role-playing [18, 25–27]. This approach could be adopted as the universal standard approach for setting activity standards.

While analyzing and interpreting WISN results was considered easy, ensuring that the results of the exercise are accepted and implemented is harder. This is partly because, in the real life of policy-making, decision-making is largely a political than a technical process that simply relies on scientific evidence even when it is available and of good quality. Implementation of WISN recommendations also has financial implications that are not catered for in most countries which have not yet adopted the use the methodology. Gagliardi et al. [28] state that the decision-making process is influenced by several factors, such as the beliefs and values of the decision makers, the timing of the evidence, the economic situation pertaining, and politics, and not only evidence. Therefore, to promote use of WISN study recommendations, WISN users need to consider the factors that influence decision-making in their country, advocate for enabling staffing policies, and consider providing alternative implementation scenarios, or phasing implementation of WISN recommendations to make implementation feasible.

### Adapting approaches from other methods

There are opportunities to learn from other methods of determining HRH requirements such as the Workforce Optimization Model (WFOM) [29] and the HRH Optimization Tool for Anti-retroviral Therapy (HOT4ART) model [30] to address some of the difficulties highlighted about the WISN methodology. Both the WFOM and HOT4ART models use the same theoretical assumptions as the WISN methodology but differ in how the activity standards are set and how the time spent on support and additional activities is managed.

While activity standards in the WISN are set only basing on discussions with the expert working groups, activity standards for the WFOM and HO4ART use a mixed-methods approach and complement the expert discussions with observations. Both the WFOM and HOT4ART account for the time spent on support and additional activities by determining the “patient facing time”, the time that a health worker spends providing health care directly to a client [30]. If these approaches were adopted for the WISN, too, they would significantly ease its implementation by negating the step of calculating allowance standards which is considered the most complex step of the WISN.

### Harnessing technologies

Automation of the WISN in some countries was highlighted as a key factor that eased WISN implementation. This is supported by current literature, which shows that technology presents an opportunity for tremendous innovation in health [31]. For example, the WFOM enables automated data entry and some WISN users have already used technology to overcome key implementation challenges. For example, in Namibia, McQuide et al. [25] developed software add-ins that enabled automated data entry into the WISN Software to overcome the challenge of laborious data entry. This significantly eased WISN implementation. However, while automation eases data entry, is cost-effective, and minimizes data entry errors, it requires extensive data validation, because small errors in an automated capture system can cause significant problems in the data sets [32, 33]. To make further harnessing of the WISN software possible, making it open-source and accessible to the international community of software developers and WISN-users’ needs to be considered. Several software programs already benefit from this approach.

### Advocating for the WISN methodology

Task-shifting, data quality issues, and lack of enabling staffing policies were some of the cross-cutting challenges highlighted by this study. These findings agree with literature which highlights the need to consider task-shifting as an alternative practice scope scenario so that activity standards are set for what people are doing, rather than what they are supposed to be doing [34–36]. This could be achieved by updating the WISN Software to generate results with a task-sharing scenario and a scenario without task-shifting learning from the HOT4ART model which generates results with different task-sharing scenarios. Improving the quality of workload and staffing data and ensuring interoperability of data systems is another priority area that needs to be worked on to ease WISN implementation. Finally, advocating for the use of the flexible WISN methodology instead of the rigid and fixed “staff establishments” and “staffing norms” is an important approach to addressing staffing shortages. However, this would require the WHO supporting the systematic documentation and targeted dissemination of research evidence that demonstrates the cost-effectiveness of the WISN methodology, and its positive impact on HRH availability, coverage of health care services and health outcomes [37].

## Study limitations

One possible limitation of this study is a possibility of bias given the fact that all the study participants were already experts with the methodology and likely to rate it as “easy”. However, using experts was rendered inevitable, since the study required respondents familiar with the methodology to assess it objectively. We addressed this potential limitation using expert users who had WISN experience of varying duration. While 50% of the respondents had conducted two or more WISN studies, the other 50% had conducted only one WISN study. In addition, 61% had more than 2 years of WISN experience, 39% of the respondents had 2 years of experience or less.

## Conclusions

This study highlights the difficulties that a new WISN user should anticipate when applying the methodology, and the strategies/innovations for addressing them. Therefore, the findings can serve as a useful reference for new users and could spur increased use of the methodology. The study also reveals several approaches for easing the design, implementation, and adoption of the WISN methodology. Based on these findings WHO’s WISN Thematic Working Group could consider revising the WISN methodology and its related tools to further ease its wider adoption and application. The WHO could also consider leading focused advocacy for its member states to adopt and use the WISN for harmonized and comparable international health workforce planning.

## Supplementary Information

**Additional file 1.** Detailed results for each Delphi iteration (1-3).

## Data Availability

The data sets supporting the conclusions of this article are included within the article and its Additional file 1.

## Abbreviations

AWT: Available working time
CAF: Category allowance factors
HOT4ART: HRH Optimization Tool for Anti-retroviral Therapy
HRH: Human Resources for Health
WFOM: Workforce Optimization Model
WHO: The World Health Organization
WISN: Workload Indicators of Staffing Need
